# Distinct Systemic Inflammatory Signatures in Women with Endometriosis and Infertility Without Endometriosis: Implications for Assisted Reproduction

**DOI:** 10.3390/medicina62091641

**Published:** 2026-08-27

**Authors:** Luana Ghilea (Seleș), Laura Maghiar, Viorela Romina Murvai, Goman Marius, Alin Bodog, Carmen Anca Huniadi

**Affiliations:** 1 Department of Surgical Sciences, Obstetrics and Gynecology, Faculty of Medicine and Pharmacy, University of Oradea, 410087 Oradea, Romania; laura.maghiar@uoradea.ro (L.M.); alin.bodog@didactic.uoradea.ro (A.B.);; 2 Doctoral School of Biological and Biomedical Sciences, University of Oradea, 1 University Street, 410087 Oradea, Romania; 3 Department of Obstetrics and Gynecology, Calla—Infertility Diagnostic and Treatment Center, Constantin A. Rosetti Street, 410103 Oradea, Romania; dr.rominamurvai@gmail.com (V.R.M.); marius_goman28@yahoo.com (G.M.); 4 Department of Embryology, Calla—Infertility Diagnostic and Treatment Center, Constantin A. Rosetti Street, 410103 Oradea, Romania

**Keywords:** endometriosis, infertility, inflammation

## Abstract

*Background and Objectives:* Endometriosis is a chronic inflammatory disease associated with impaired fertility, but the extent to which its systemic inflammatory profile differs from that observed in infertile women without endometriosis remains incompletely understood. This study aimed to characterize and compare the systemic inflammatory profiles of women with endometriosis, infertile women without endometriosis, and healthy controls by evaluating serum interleukin-6 (IL-6), tumor necrosis factor-alpha (TNF-α), and high-sensitivity C-reactive protein (hs-CRP). In addition, the study explored the IL-6/TNF-α ratio as an indicator of inflammatory balance and evaluated its relationship with ovarian reserve. *Materials and Methods:* This observational comparative study included 87 women divided into three groups: healthy controls (*n* = 29), women with endometriosis (*n* = 29), and infertile women without endometriosis (*n* = 29). Serum IL-6, TNF-α, and hs-CRP concentrations were determined specifically for the present study, whereas the corresponding clinical and embryological data were retrieved from medical records. Group comparisons were performed using the Kruskal–Wallis test followed by Dunn’s post hoc test with Holm correction. The IL-6/TNF-α ratio was calculated as an exploratory indicator of inflammatory balance. Associations between inflammatory biomarkers and anti-Müllerian hormone (AMH) concentrations were assessed using Spearman’s correlation analysis. *Results:* Significant differences in serum inflammatory biomarkers were observed among the three groups. IL-6 concentrations were significantly increased in both women with endometriosis and infertile women without endometriosis compared with healthy controls, whereas TNF-α concentrations were significantly elevated only in infertile women without endometriosis compared with both healthy controls and women with endometriosis (adjusted *p* < 0.001 for both comparisons). Differences in hs-CRP were more modest but remained significant overall. Women with endometriosis exhibited a significantly higher IL-6/TNF-α ratio than infertile women without endometriosis, suggesting distinct inflammatory patterns between these conditions. No significant correlations were identified between circulating inflammatory biomarkers and AMH concentrations. *Conclusions:* Women with endometriosis and infertile women without endometriosis exhibit distinct systemic inflammatory profiles rather than simply different levels of inflammation. The IL-6/TNF-α ratio may provide additional information regarding the balance of inflammatory pathways beyond the isolated evaluation of individual cytokines. Larger prospective studies integrating systemic and local inflammatory biomarkers with assisted reproductive outcomes are warranted to validate these findings and further explore their clinical relevance.

## 1. Introduction

Endometriosis is widely recognized as a chronic, estrogen-dependent inflammatory disease with important consequences for female fertility and reproductive health. Beyond the mechanical effects of pelvic adhesions, endometriomas, altered tubo-ovarian anatomy, or previous surgical interventions, endometriosis may impair reproductive potential through persistent inflammation, immune dysregulation, oxidative stress, an altered follicular environment, and impaired endometrial receptivity [1,2].

Inflammation plays a central role in the pathophysiology of endometriosis by promoting lesion establishment, angiogenesis, immune-cell recruitment, and tissue remodeling. These processes extend beyond the pelvic cavity and may contribute to systemic immune activation, thereby influencing reproductive function. In women undergoing assisted reproductive treatment, systemic inflammatory alterations have been proposed as one of the mechanisms contributing to impaired ovarian function, altered oocyte competence, and reduced endometrial receptivity, although the biological pathways involved remain incompletely understood [2,3].

Among the inflammatory mediators investigated in endometriosis, interleukin-6 (IL-6), tumor necrosis factor-alpha (TNF-α), and high-sensitivity C-reactive protein (hs-CRP) are of particular interest. IL-6 is a pleiotropic cytokine involved in immune activation, inflammatory signaling, angiogenesis, and tissue remodeling. TNF-α is a key pro-inflammatory cytokine associated with macrophage activation, inflammatory amplification, and altered cellular communication within the reproductive microenvironment. hs-CRP, although less specific, reflects low-grade systemic inflammation and may provide complementary information regarding the overall inflammatory status of women with reproductive disorders [1,3,4].

Previous studies have demonstrated increased circulating concentrations of IL-6, TNF-α, and other inflammatory mediators in women with endometriosis; however, their diagnostic performance has been inconsistent, and considerable overlap exists with other inflammatory and reproductive conditions [1,2,4]. Consequently, individual inflammatory biomarkers are unlikely to provide sufficient diagnostic accuracy when interpreted in isolation. Instead, growing evidence suggests that systemic inflammation should be considered a complex biological process involving multiple interacting pathways rather than single-cytokine alterations.

In the context of assisted reproduction, systemic inflammatory status has also attracted increasing attention. Elevated baseline hs-CRP concentrations have been associated with reduced reproductive success following in vitro fertilization, suggesting that low-grade systemic inflammation may influence reproductive competence even in the absence of overt inflammatory disease [5]. Nevertheless, whether women with endometriosis exhibit inflammatory characteristics that differ from those observed in infertile women without endometriosis remains insufficiently understood.

Although IL-6, TNF-α, and hs-CRP have been extensively investigated in endometriosis and infertility, most studies have evaluated these biomarkers individually or primarily focused on their diagnostic performance. Comparatively little attention has been given to the possibility that women with endometriosis and infertile women without endometriosis may exhibit distinct systemic inflammatory profiles rather than simply different concentrations of individual cytokines. Identifying such inflammatory patterns may improve our understanding of the biological heterogeneity of reproductive disorders and contribute to a more comprehensive interpretation of systemic inflammation.

Therefore, the present study aimed to characterize and compare the systemic inflammatory profiles of women with endometriosis, infertile women without endometriosis, and healthy controls by evaluating three circulating inflammatory biomarkers (IL-6, TNF-α, and hs-CRP). In addition, the study explored whether the relative balance between IL-6 and TNF-α, assessed through the IL-6/TNF-α ratio, could reveal distinct inflammatory patterns beyond the isolated evaluation of individual biomarkers. Exploratory analyses were also performed to investigate the relationship between systemic inflammatory biomarkers and ovarian reserve, as reflected by anti-Müllerian hormone (AMH) concentrations. By focusing on integrated inflammatory profiles rather than isolated cytokine alterations, this study aims to contribute to a better biological characterization of women with endometriosis and infertility and to support future approaches toward personalized reproductive medicine.

Although systemic inflammation has been extensively investigated in endometriosis, it remains unclear whether women with endometriosis exhibit a distinct inflammatory phenotype or simply share the systemic inflammatory characteristics associated with infertility itself. Clarifying these differences may improve the biological understanding of reproductive disorders and help identify disease-specific inflammatory signatures.

## 2. Materials and Methods

### 2.1. Study Design and Setting

This observational comparative study included women who underwent in vitro fertilization/intracytoplasmic sperm injection (IVF/ICSI) procedures. Serum inflammatory biomarkers were measured specifically for the present study, while the corresponding clinical and embryological data were retrieved from medical records. The study focused on the evaluation of three serum inflammatory biomarkers—interleukin-6 (IL-6), tumor necrosis factor-alpha (TNF-α), and high-sensitivity C-reactive protein (hs-CRP)—in women with endometriosis, infertile women without endometriosis, and healthy controls. The study was conducted in accordance with the principles of the Declaration of Helsinki. The research protocol was approved by the Institutional Review Board of the Calla IVF Center (protocol code 3678/A, 5 January 2026). All patient data were anonymized before analysis, and confidentiality was maintained throughout the study.

### 2.2. Study Population

The study population consisted of women of reproductive age evaluated in the context of infertility and assisted reproductive technology (IVF/ICSI). According to their clinical diagnosis, participants were allocated into three study groups:

Endometriosis group: women with a confirmed diagnosis of endometriosis who underwent IVF/ICSI treatment.

Infertility without endometriosis group: women undergoing IVF/ICSI for infertility-related indications other than endometriosis and without clinical, imaging, or surgical evidence of the disease.

Control group: women without endometriosis or infertility who served as healthy controls for the evaluation of circulating inflammatory biomarkers.

The diagnosis of endometriosis was established based on clinical history, transvaginal ultrasound findings, magnetic resonance imaging when available, previous laparoscopic findings, and/or histopathological confirmation. Patients with ovarian endometriomas were included when the diagnosis was supported by characteristic imaging findings or previous surgical confirmation. Women included in the infertility-without-endometriosis group had no clinical, imaging, or surgical evidence of endometriosis and underwent IVF/ICSI for infertility-related indications other than endometriosis, including male factor infertility, tubal factor infertility, unexplained infertility, or other non-endometriosis-related causes.

### 2.3. Inclusion and Exclusion Criteria

Women of reproductive age were eligible for inclusion. Only participants with available clinical data and serum measurements of IL-6, TNF-α, and hs-CRP were included in the analysis. Women in the endometriosis group had a confirmed diagnosis established on the basis of clinical evaluation, imaging findings, previous laparoscopic surgery, and/or histopathological confirmation. The infertility without endometriosis group included women undergoing IVF/ICSI for infertility-related indications other than endometriosis and without clinical, imaging, or surgical evidence of the disease. The control group consisted of healthy women without endometriosis or infertility who served as controls for the evaluation of circulating inflammatory biomarkers.

Equal group sizes (*n* = 29 per group) were intentionally selected to ensure a balanced comparative design across the three study groups; no formal a priori sample size calculation was performed.

Patients were excluded if clinical data or serum biomarker measurements (IL-6, TNF-α, or hs-CRP) were unavailable. Additional exclusion criteria included the presence of acute infection at the time of blood sampling; chronic inflammatory or autoimmune diseases that could significantly influence systemic inflammatory markers; recent use of systemic corticosteroids, anti-inflammatory drugs, or immunomodulatory therapy; active malignancy; and severe systemic disease.

### 2.4. Statistical Analysis

Statistical analysis was performed using IBM SPSS Statistics version 29.0 (IBM Corp., Armonk, NY, USA).

The distribution of continuous variables was assessed using the Shapiro–Wilk test. Continuous variables are presented as mean ± standard deviation (SD) and median with interquartile range (IQR), as appropriate according to data distribution. Categorical variables are presented as frequencies and percentages.

Comparisons of continuous variables among the three study groups were performed using the Kruskal–Wallis test because most inflammatory biomarkers were non-normally distributed. When the overall test was statistically significant, pairwise post hoc comparisons were performed using Dunn’s test with Holm adjustment for multiple comparisons.

To further characterize the inflammatory profile of the study groups, the serum IL-6/TNF-α ratio was calculated for each participant and analyzed using the same non-parametric approach.

Associations between serum inflammatory biomarkers and anti-Müllerian hormone (AMH) concentrations were evaluated using Spearman’s rank correlation coefficient. These analyses were considered exploratory because AMH measurements were available only for a subset of participants.

A two-sided *p*-value < 0.05 was considered statistically significant.

## 3. Results

### 3.1. Baseline Serum Inflammatory Profile of the Study Groups

A total of 87 women were included in the study, comprising 29 healthy controls, 29 women diagnosed with endometriosis, and 29 infertile women without endometriosis. Serum concentrations of IL-6, TNF-α, and hs-CRP were determined in all participants to evaluate the systemic inflammatory profile associated with endometriosis and infertility. Together, these biomarkers were used to characterize the systemic inflammatory profile of each study group.

The descriptive statistics of the inflammatory biomarkers are presented in Table 1. Women with infertility without endometriosis exhibited the highest mean serum concentrations of IL-6 (16.19 ± 21.14 pg/mL) and TNF-α (70.52 ± 32.34 pg/mL) among the three study groups. Patients with endometriosis showed intermediate IL-6 concentrations (7.08 ± 7.31 pg/mL) compared with healthy controls (3.57 ± 5.10 pg/mL). Mean TNF-α concentrations were comparable between the endometriosis (17.90 ± 22.41 pg/mL) and control (16.29 ± 14.95 pg/mL) groups. Serum hs-CRP concentrations were 1.72 ± 2.87 mg/L in healthy controls, 2.27 ± 4.49 mg/L in women with endometriosis, and 2.74 ± 2.79 mg/L in infertile women without endometriosis.

**Figure 1:**
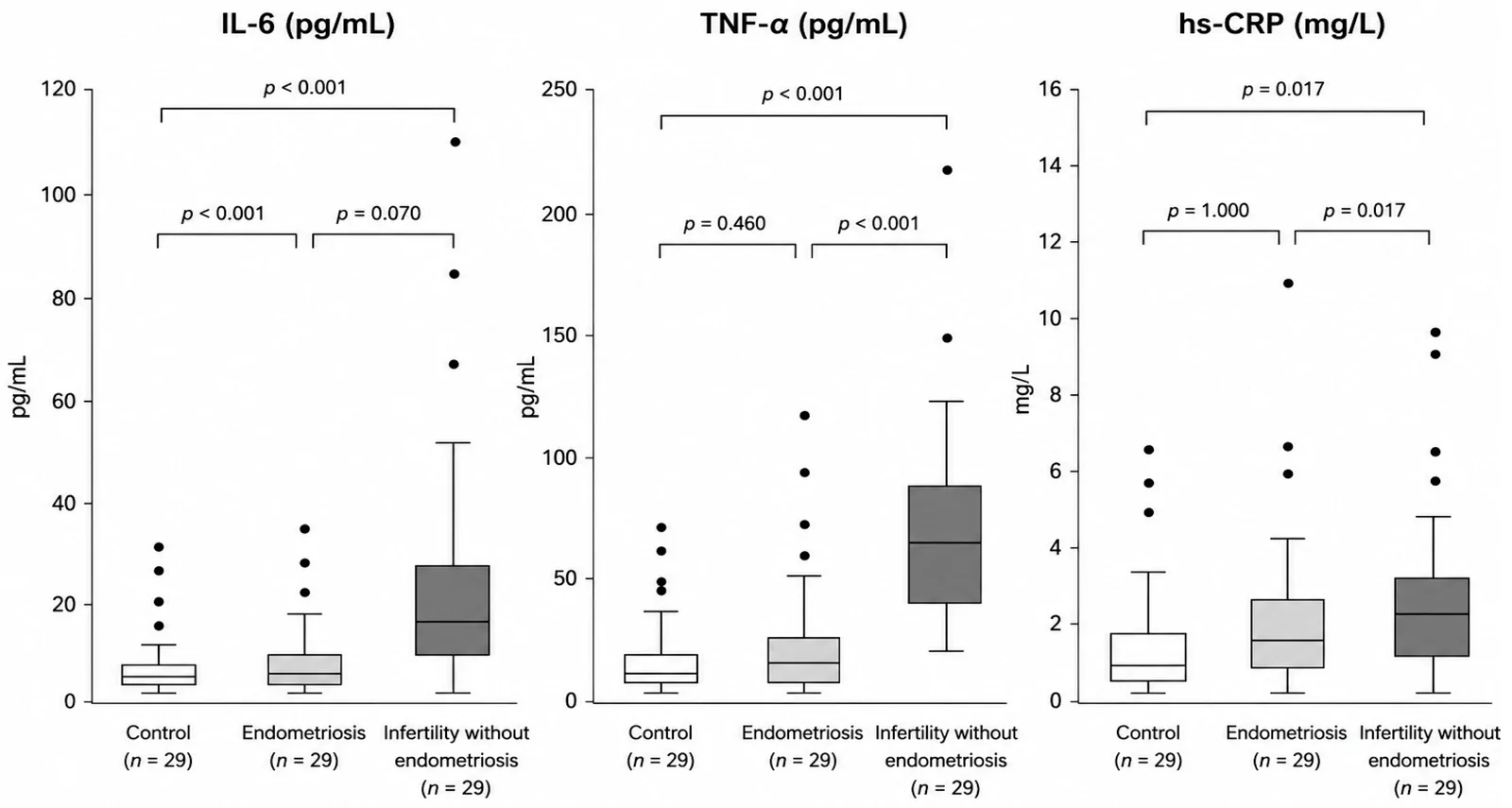
Distribution of serum IL-6, TNF-α, and hs-CRP concentrations across the three study groups. Boxplots display the median (horizontal line), interquartile range (box), 1.5 × interquartile range (whiskers), and outliers (●). Group comparisons were performed using the Kruskal–Wallis test followed by Dunn’s post hoc test with Holm correction for multiple comparisons.

Figure 1 illustrates the distribution of serum inflammatory biomarkers across the three study groups, complementing the descriptive statistics presented in Table 1 and highlighting the differences in biomarker profiles.

### 3.2. Comparison of Serum IL-6 Levels Among the Study Groups

Serum IL-6 concentrations differed significantly among the three study groups (Kruskal–Wallis test, *p* < 0.001). The highest IL-6 levels were observed in infertile women without endometriosis, with a median concentration of 8.13 pg/mL (IQR: 3.21–12.20). Women with endometriosis presented intermediate values, with a median of 4.59 pg/mL (IQR: 2.43–7.99), whereas the control group had the lowest IL-6 concentrations, with a median of 1.57 pg/mL (IQR: 1.50–3.35).

Pairwise comparisons showed that IL-6 concentrations were significantly higher in women with endometriosis than in healthy controls (adjusted *p* < 0.001). Infertile women without endometriosis also had significantly higher IL-6 levels than the control group (adjusted *p* < 0.001). However, the difference between the endometriosis and infertility-without-endometriosis groups did not reach statistical significance (adjusted *p* = 0.070).

### 3.3. Comparison of Serum TNF-α Concentrations Among the Study Groups

Serum TNF-α concentrations differed significantly among the three study groups (Kruskal–Wallis test, *p* < 0.001). The overall difference was primarily driven by the infertility-without-endometriosis group, which exhibited the highest circulating TNF-α concentrations. In contrast, women with endometriosis showed serum TNF-α levels comparable to those observed in healthy controls.

Post hoc pairwise comparisons confirmed significantly higher TNF-α concentrations in infertile women without endometriosis than in both healthy controls (adjusted *p* < 0.001) and women with endometriosis (adjusted *p* < 0.001). No statistically significant difference was identified between the endometriosis and control groups (adjusted *p* = 0.460).

These findings indicate that the significant overall difference in circulating TNF-α concentrations was attributable to the infertility-without-endometriosis group rather than to women with endometriosis.

### 3.4. Comparison of Serum Hs-CRP Levels Among the Study Groups

Serum hs-CRP concentrations differed significantly among the three study groups (Kruskal–Wallis test, *p* = 0.008). The highest hs-CRP concentrations were observed in infertile women without endometriosis, followed by women with endometriosis, whereas healthy controls exhibited the lowest serum levels.

Pairwise comparisons demonstrated significantly higher hs-CRP concentrations in infertile women without endometriosis than in healthy controls (*p* = 0.017) and women with endometriosis (*p* = 0.017). No statistically significant difference was observed between women with endometriosis and healthy controls (*p* = 1.000).

### 3.5. IL-6/TNF-α Ratio as a Marker of Distinct Inflammatory Patterns

To explore whether the study groups were characterized by distinct cytokine patterns rather than only differences in absolute biomarker concentrations, the serum IL-6/TNF-α ratio was calculated for each participant. This ratio differed significantly among the three study groups (Kruskal–Wallis test, *p* < 0.001).

Women with endometriosis exhibited the highest median IL-6/TNF-α ratio, at 0.75 (IQR: 0.22–1.08). In contrast, infertile women without endometriosis showed a markedly lower median ratio of 0.10 (IQR: 0.06–0.46), despite presenting the highest absolute concentrations of both inflammatory cytokines. Healthy controls had a median ratio of 0.21 (IQR: 0.15–0.35) Table 2.

Post hoc pairwise analysis showed that the IL-6/TNF-α ratio was significantly higher in women with endometriosis than in healthy controls (adjusted *p* = 0.004) and in infertile women without endometriosis (adjusted *p* = 0.004). No statistically significant difference was observed between healthy controls and infertile women without endometriosis (adjusted *p* = 0.100) Table 3.

These findings support the hypothesis that endometriosis and infertility without endometriosis may be associated with different systemic inflammatory signatures.

**Table 2:** Serum IL-6/TNF-α ratio among the study groups.

Study Group	*n*	Mean ± SD	Median (IQR)
Control	29	0.24 ± 0.12	0.21 (0.15–0.35)
Endometriosis	29	1.01 ± 1.10	0.75 (0.22–1.08)
Infertility without endometriosis	29	0.71 ± 2.46	0.10 (0.06–0.46)

Overall comparison: Kruskal–Wallis test, *p* < 0.001.

The wide dispersion of the IL-6/TNF-α ratio observed in the infertility-without-endometriosis group (mean ± SD: 0.71 ± 2.46) reflected the presence of extreme values and a markedly skewed distribution. Therefore, group comparisons were based on nonparametric rank-based methods and median (IQR) values, which are less sensitive to outliers.

**Table 3:** Pairwise comparisons of the serum IL-6/TNF-α ratio.

Comparison	Adjusted *p*-Value (Holm)
Control vs. Endometriosis	0.004
Control vs. Infertility without endometriosis	0.100
Endometriosis vs. Infertility without endometriosis	0.004

### 3.6. Exploratory Association Between Serum Inflammatory Biomarkers and Ovarian Reserve (AMH)

To explore the potential reproductive relevance of the systemic inflammatory profile, correlations among serum IL-6, TNF-α, hs-CRP, and anti-Müllerian hormone (AMH) concentrations were evaluated in women with endometriosis and in those without endometriosis. Because AMH measurements were available for only a subset of participants, these analyses were considered exploratory.

In women with endometriosis, no statistically significant correlations were observed between AMH and IL-6 (Spearman’s ρ = −0.225, *p* = 0.459), TNF-α (ρ = 0.190, *p* = 0.535), or hs-CRP (ρ = 0.016, *p* = 0.957). Similarly, no statistically significant associations were identified in infertile women without endometriosis. Although TNF-α demonstrated a moderate inverse correlation with AMH in this group (ρ = −0.399), the association did not reach statistical significance (*p* = 0.177) Table 4.

Overall, these findings suggest that systemic inflammatory biomarker concentrations were not significantly associated with ovarian reserve, as reflected by AMH levels, within the available study sample. Nevertheless, the limited number of participants with available AMH measurements warrants caution in interpreting these exploratory analyses.

**Table 4:** Spearman correlations between serum inflammatory biomarkers and AMH.

Study Group	Correlation	Sample Size (*n*)	Spearman’s ρ	*p*-Value
Endometriosis	IL-6 vs. AMH	12	−0.225	0.459
Endometriosis	TNF-α vs. AMH	13	0.190	0.535
Endometriosis	hs-CRP vs. AMH	12	0.016	0.957
Infertility without endometriosis	IL-6 vs. AMH	11	0.153	0.653
Infertility without endometriosis	TNF-α vs. AMH	13	−0.399	0.177
Infertility without endometriosis	hs-CRP vs. AMH	11	0.043	0.899

## 4. Discussion

The present study demonstrated that women with endometriosis and infertile women without endometriosis exhibit distinct systemic inflammatory profiles rather than simply different degrees of inflammation. Although both groups showed evidence of systemic inflammatory activation, the relative behavior of IL-6, TNF-α, and hs-CRP differed, suggesting that these reproductive conditions may involve different inflammatory mechanisms. These findings indicate that evaluating multiple circulating biomarkers provides a more comprehensive characterization of systemic inflammation than interpreting individual cytokines in isolation.

Among the biomarkers evaluated, IL-6 was consistently elevated in both pathological groups compared with healthy controls, consistent with prior evidence identifying IL-6 as an important mediator of chronic inflammation in endometriosis [6]. Elevated circulating IL-6 has been associated with persistent immune activation and disease severity, although its diagnostic value remains limited, as similar elevations have also been reported in other inflammatory and reproductive disorders [7,8]. Therefore, IL-6 appears to reflect systemic inflammatory activity rather than representing a disease-specific biomarker.

Higher IL-6 concentrations were observed in infertile women without endometriosis than in those with endometriosis; however, this difference did not reach statistical significance (adjusted *p* = 0.070). The observed numerical difference suggests a possible trend that should be interpreted with caution and further investigated in larger cohorts.

This observation is consistent with the recognized biological heterogeneity of infertility and further supports the concept that inflammatory biomarkers should be interpreted within the broader clinical context rather than individually [9,10,11].

The pattern of serum TNF-α observed in the present study differed from that of IL-6. While IL-6 concentrations were increased in both pathological groups, elevated TNF-α levels were observed only in infertile women without endometriosis. In contrast, women with endometriosis showed serum TNF-α concentrations comparable to those of healthy controls (adjusted *p* = 0.460). This finding differs from some previous studies reporting increased circulating TNF-α levels in women with endometriosis [12,13,14,15,16]; however, published findings remain inconsistent. Our results suggest that systemic TNF-α may not reliably distinguish women with endometriosis from healthy controls and that elevated circulating TNF-α may be more closely related to specific infertility-associated inflammatory mechanisms than to endometriosis itself. The differences observed across studies are likely multifactorial and may reflect variations in patient selection, disease stage, infertility etiology, timing of blood sampling, and analytical methodology [12,13,14,15,16]. Moreover, systemic TNF-α concentrations may not accurately reflect the inflammatory processes occurring within endometriotic lesions or the peritoneal microenvironment. Consequently, the absence of elevated circulating TNF-α in our endometriosis cohort should not be interpreted as evidence of limited inflammatory activity. Conversely, the marked increase observed in infertile women without endometriosis may reflect inflammatory mechanisms associated with infertility itself or with other underlying clinical conditions not specifically evaluated in the present study. These findings should therefore be interpreted cautiously and confirmed in larger, well-characterized cohorts [17,18,19,20].

Serum hs-CRP concentrations also differed significantly among the study groups, although the observed differences were less pronounced than those identified for IL-6 and TNF-α. As a marker of low-grade systemic inflammation, hs-CRP lacks disease specificity but provides complementary information regarding the overall inflammatory status of women with reproductive disorders. Our findings are consistent with previous studies suggesting that systemic inflammatory activation extends beyond alterations in individual cytokines and may contribute to reproductive dysfunction [21].

An interesting observation was the significantly higher IL-6/TNF-α ratio in women with endometriosis compared with infertile women without endometriosis. Previous studies have primarily evaluated individual cytokines, whereas our findings suggest that assessing the balance between inflammatory mediators may provide additional information on systemic inflammatory responses. Because this analysis was exploratory, the IL-6/TNF-α ratio cannot yet be considered a validated biomarker. Its potential clinical value should be investigated in larger prospective studies [22,23,24,25].

The exploratory analyses did not identify significant correlations between circulating inflammatory biomarkers and AMH concentrations. Although these analyses were limited by the availability of AMH measurements in only a subset of participants, the findings suggest that systemic inflammatory activity was not directly associated with ovarian reserve within the present cohort. Because ovarian reserve represents only one aspect of female reproductive function, additional studies integrating inflammatory biomarkers with clinical and reproductive outcomes are needed to clarify these relationships [26,27,28,29,30].

Several limitations should be acknowledged. First, the relatively small sample size may have limited the statistical power, particularly for the exploratory analyses involving AMH. In addition, only three circulating inflammatory biomarkers were evaluated, whereas the inflammatory pathways involved in endometriosis are considerably more complex. Finally, serum biomarker concentrations may not fully reflect the local inflammatory environment within the peritoneal cavity, follicular fluid, or endometrium. Despite these limitations, the study has important strengths. The inclusion of three clinically distinct groups enabled direct comparison between inflammatory alterations associated with endometriosis and those observed in infertility without endometriosis. Furthermore, the combined evaluation of IL-6, TNF-α, hs-CRP, and the exploratory IL-6/TNF-α ratio provided a broader characterization of systemic inflammatory profiles than the assessment of individual biomarkers alone. Future prospective studies including larger cohorts, additional inflammatory mediators, and detailed reproductive outcomes are required to validate these findings and further define the role of systemic inflammatory profiling in women with endometriosis [31].

## 5. Conclusions

This study demonstrates that women with endometriosis and infertile women without endometriosis exhibit distinct systemic inflammatory profiles rather than simply different degrees of systemic inflammation. Although both conditions were associated with inflammatory activation, the relative distribution of IL-6, TNF-α, and hs-CRP differed substantially, suggesting that different biological mechanisms may underlie these reproductive disorders.

Among the evaluated biomarkers, IL-6 was increased in both pathological groups, whereas elevated TNF-α concentrations were predominantly observed in infertile women without endometriosis. In addition, the exploratory analysis of the IL-6/TNF-α ratio indicated that the relative balance between inflammatory mediators may provide complementary information beyond the isolated assessment of individual cytokines, supporting the concept of distinct inflammatory signatures.

No significant associations were identified between circulating inflammatory biomarkers and AMH concentrations; however, these analyses were exploratory and based on small subsamples and therefore may have been underpowered to detect modest associations. These findings should consequently be interpreted with caution and require confirmation in larger cohorts.

Overall, our findings support the use of an integrated inflammatory profiling approach to improve the biological characterization of women with endometriosis and infertility. Future prospective studies including larger cohorts, comprehensive clinical phenotyping, local and systemic inflammatory biomarkers, and assisted reproductive outcomes are needed to validate these inflammatory signatures and determine their potential clinical value for patient stratification and personalized reproductive medicine.

## Figures and Tables

**Table 1:** Baseline serum inflammatory biomarkers in the study population.

Biomarker	Control (*n* = 29) Mean ± SD	Control Median (IQR)	Infertility Without Endometriosis (*n* = 29) Mean ± SD	Infertility Without Endometriosis Median (IQR)	Endometriosis (*n* = 29) Mean ± SD	Endometriosis Median (IQR)
IL-6 (pg/mL)	3.57 ± 5.10	1.57 (1.50–3.35)	16.19 ± 21.14	8.13 (3.21–12.20)	7.08 ± 7.31	4.59 (2.43–7.99)
TNF-α (pg/mL)	16.29 ± 14.95	7.60 (6.40–34.30)	70.52 ± 32.34	76.60 (50.90–93.40)	17.90 ± 22.41	7.40 (5.70–10.90)
hs-CRP (mg/L)	1.72 ± 2.87	0.43 (0.30–1.74)	2.74 ± 2.79	2.60 (0.74–3.03)	2.27 ± 4.49	0.49 (0.29–1.43)

Note: Data are presented as mean ± standard deviation (SD) and median (interquartile range, IQR).

## Data Availability

The data presented in this study are available from the corresponding author upon reasonable request. The data are not publicly available because they contain information that could compromise participant privacy and are subject to ethical restrictions.
